# No evidence of association between human cytomegalovirus infection and papillary thyroid cancer

**DOI:** 10.1186/1477-7819-12-41

**Published:** 2014-02-21

**Authors:** Tung-Sun Huang, Jie-Jen Lee, Shih-Ping Cheng

**Affiliations:** 1 Department of Surgery, Mackay Medical College and Mackay Memorial Hospital, No. 92, Sec 2, Chung-Shan North Road, Taipei 10449, Taiwan; 2 Mackay Junior College of Medicine, Nursing, and Management, No. 92, Sheng-Ching Road, Peitou, Taipei 11260, Taiwan; 3 Department of Pharmacology and Graduate Institute of Medical Sciences, Taipei Medical University, No. 250, Wu-Hsing Street, Taipei 11031, Taiwan

**Keywords:** Cytomegalovirus, BRAF, Papillary thyroid cancer

## Abstract

**Background:**

Human cytomegalovirus (CMV) has been detected in the thyroid gland and thyroid tumors. CMV infection may activate the mitogen-activated protein kinase pathway, of which aberrant activation is frequently associated with BRAF mutation in papillary thyroid cancer.

**Methods:**

A total of 45 paired tumorous and adjacent non-neoplastic tissue samples, including 5 follicular adenoma and 40 papillary thyroid cancer, were obtained during thyroidectomy. BRAF mutational status was determined using direct sequencing. The presence of CMV DNA was determined using conventional PCR and quantitative real-time PCR. CMV protein in the tissue samples were evaluated with Western blot analysis.

**Results:**

BRAF mutation was identified in the cancerous part of 31 (78%) papillary thyroid cancers. Papillary cancer with BRAF mutation was significantly associated with a larger tumor size (*P* = 0.045), extrathyroidal invasion (*P* = 0.012), lymph node metastasis (*P* = 0.008), and a higher TNM stage (*P* = 0.044). CMV DNA and protein were not detected in any studied samples.

**Conclusions:**

Our results suggest no association between CMV infection and papillary thyroid cancer.

## Background

Differentiated thyroid cancer arising from the follicular epithelium is the most common endocrine malignancy, and papillary thyroid cancer accounts for the majority of differentiated thyroid cancers [1]. Given the fact that the prevalence of familial non-medullary thyroid cancer is only about 5% [2], differentiated thyroid cancer is mostly sporadic. The only established epidemiological factors in association with thyroid cancer are ionizing radiation and iodine deficiency [3]. Nonetheless, most patients diagnosed to have thyroid cancer do not have these predisposing factors. Therefore, the mechanisms underlying thyroid cancer development are still poorly defined.

Many genetic and epigenetic alterations have been implicated in the pathogenesis of thyroid cancer. The v-raf murine sarcoma viral oncogene homolog B (BRAF) mutation is the most common genetic alteration in papillary thyroid cancer [1]. BRAF activates the mitogen-activated protein kinase (MAPK) pathway and plays an important role in regulating cellular differentiation, proliferation, and survival [4]. Oncogenic BRAF may trigger a proinflammatory program in thyroid epithelial cells [5]. Recently, we demonstrated that preoperative blood neutrophil-to-lymphocyte ratio, a surrogate marker for systemic inflammation, correlated with tumor size in differentiated thyroid cancer [6]. In this context, it remains controversial whether the inflammation is the cause or consequence in the tumorigenesis of thyroid cancer.

Human cytomegalovirus (CMV) is a member of the Herpesviridae family of viruses. Patients with CMV infection have variable clinical manifestations, from no disease in healthy hosts to congenital CMV syndrome in neonates [7]. Meningoencephalitis, retinitis, pneumonitis, myocarditis, hepatitis, enterocolitis, and disseminated disease may be seen in immunocompromised patients and transplant recipients. After a primary infection, which is generally asymptomatic in immunocompetent persons, CMV establishes latency and persists in its host. CMV seroprevalence increases with age. In most studies, seroprevalence reached 60% or more in individuals older than 50 years [8]. Recently, a new entity of infection, called “microinfection”, has been used to describe the low levels of CMV infection found in inflammatory diseases and certain cancers [9]. Through mechanisms involving oncogenic transformation, oncomodulation, and tumor cell immune evasion, CMV infection has been implicated in several cancer types [10]. It has been shown that CMV infection may induce a prosurvival state of latently infected cells via activation of the MAPK signaling pathway [11].

Sensitive techniques have been developed to detect the presence of CMV genome or antigens in specific tissues. In a small series, CMV was the only virus present in thyroid tumors [12]. In another study examining herpes virus tissue distribution, CMV was detected in the thyroid gland in three of the eight autopsies [13]. These findings indicate that the thyroid gland is one of the reservoirs of latent human CMV infection. Considering that the MAPK pathway is the most common genetic alteration in thyroid cancer and may be activated by CMV infection, we hypothesized that CMV infection may be involved in the pathogenesis of thyroid cancer. In the present study, we set out to examine the viral DNA and protein in papillary thyroid cancer tissues, and to correlate with the status of tumor BRAF mutation.

## Methods

### Clinical samples

Tissue samples were collected under an institutional review board-approved (Mackay Memorial Hospital 12MMHIS175) tissue procurement protocol after written informed consent was obtained. A total of 40 patients undergoing total thyroidectomy for papillary thyroid cancer and 5 patients undergoing lobectomy for follicular adenoma were included in this study. Tumor tissues from the center of the lesions and corresponding normal thyroid tissues from the contralateral lobes of the same patients were obtained. All tumor tissue samples were carefully dissected to exclude surrounding normal tissue. Tissue samples were snap frozen immediately in liquid nitrogen and stored at −80°C. The tissue diagnosis was confirmed by frozen sections.

### DNA extraction

DNA was extracted from frozen tumor tissues using the QIAamp DNA mini kit (Qiagen, Valencia, CA, USA) according to the manufacturer’s instructions. The quality of extracted DNA was examined by agarose gel electrophoresis. DNA concentrations were determined from the absorption at 260 nm. The ratio of the absorption at 260 nm to that at 280 nm was greater than 1.84 in all samples.

### Direct sequencing analysis of BRAF mutation

A fragment of 228-bp length including codon 600 of BRAF (RefSeq DNA: NM_004333) was amplified using the forward primer 5′-TGCTTGCTCTGATAGGAAAATG-3′ and the reverse primer 5′-AGCATCTCAGGGCCAAAAAT-3′. The PCR was run under standard buffer conditions as follows: 95°C for 5 minutes for one cycle; 45 cycles with denaturing at 95°C for 30 seconds, annealing at 58°C for 30 seconds, and extension at 72°C for 30 seconds. This was followed by a final extension at 72°C for 7 minutes. Amplified fragments were separated on a 2% agarose gel and visualized by ethidium bromide staining. The PCR products were column purified and subjected to sequencing reaction using the forward primer and BigDye terminator V3.1 cycle sequencing reagents (Applied Biosystems, Life Technologies, Carlsbad, CA, USA). Cycling conditions were 95°C for 5 minutes for one cycle and 95°C for 30 seconds, 55°C for 30 seconds, and 60°C for 1 minute for 45 cycles. DNA sequence was read on an ABI PRISM 3730xL DNA analyzer (Applied Biosystems), and the BRAF mutations were identified.

### Conventional PCR using custom-made primer

To determine whether viral DNA was present in the tumor samples, frozen tumor tissue specimens were examined with PCR. DNA was amplified by PCR primers specific to the CMV UL123 open reading frame (forward 5′-CGACGTTCCTGCAGACTATG-3′ and reverse 5′-TCCTCGGTCACTTGTTCAAA-3′) [14]. The expected PCR product would be 117 bp. PCR was performed in 25-μL reaction mixtures that were run for 40 cycles. PCR products were separated by electrophoresis, and amplified products were visualized on agarose gels with ethidium bromide.

### Real-time PCR assay

The artus CMV TM assay (Qiagen) targets a 105-bp region of the major immediate-early (IE) antigen. The real-time PCR was performed according to the manufacturer’s instructions. Briefly, 20 μL of processed sample were added to a working master mix, which contained 25 μL CMV TM Master, 5 μL CMV Mg-Sol, and 2 μL of CMV internal control to monitor any possible amplification inhibitors. The mixed solution was sealed with an optical adhesive film, briefly centrifuged, and amplified using the 7500 Fast Real-Time PCR System (Applied Biosystems). Cycling parameters were 95°C for 10 minutes, 45 cycles of 95°C for 15 seconds, and 55°C for 1 minute. Quantitation standards (10, 100, 1,000, and 10,000 CMV DNA copies/μL) included in the supplied kit were used to generate a standard curve in each run, allowing determination of the CMV viral load. Results were analyzed using 7500 System Sequence Detection Software version 1.4. According to the manufacturer, this PCR test has an analytical sensitivity of 0.20 copies/μL (95% probability that 0.20 copies/μL will be detected).

### Western blot analysis

Tissue lysates were prepared by treatment with lysis buffer as described previously [15]. Lysates were sonicated for 30 seconds on ice and centrifuged at 14,000 × *g* for 10 minutes at 4°C. Protein concentration was measured using the Bradford assay (Bio-Rad Laboratories, Hercules, CA, USA). For Western blotting, 50 μg of total protein were separated by electrophoresis on 10% sodium dodecyl sulfate polyacrylamide gels. Fractionated proteins were transferred to a nitrocellulose membrane, and the transfer was controlled by Ponceau staining. After transfer, the membrane was blocked with 5% skimmed milk for 30 minutes at room temperature. The proteins were probed with antibodies against CMV IE1-72 (MAB810R; Millipore, Billerica, MA, USA) and β-actin (Sigma, St. Louis, MO, USA) at 4°C overnight. The results were visualized with horseradish peroxidase-conjugated secondary antibodies (Sigma) and enhanced chemiluminescence. CMV standard lysate (The Native Antigen Company, Oxford, UK) was used as the positive control.

### Statistical analysis

Data are expressed as mean ± SD. Fisher’s exact test was used for comparison of categorical variables. The non-parametric Mann-Whitney U test was used for analysis of continuous variables. Significance of trends in stage distribution was assessed with the Cochran-Armitage test for trend. All statistical analyses were two-sided, and a *P* value <0.05 was considered statistically significant.

## Results

### Patient characteristics

Tissue samples from 5 follicular adenoma and 40 papillary thyroid cancer were used in this study after confirmation of the tissue diagnosis (Table 1). Patients with follicular adenoma underwent lobectomy. Patients with papillary thyroid cancer had total thyroidectomy and central neck lymph node dissection, with or without lateral neck dissection. The majority (36 out of 40) of papillary thyroid cancer were of classic papillary histotype, whereas four were follicular variant. Lymph node metastasis was found in 63% of the patients. More than one-third of the patients had stage III or IV disease. Six patients had pathologically confirmed Hashimoto’s thyroiditis. Thyroiditis did not correlate with tumor stage (*P* = 0.188).

**Table 1:** Clinical characteristics of the study cohort

**Features**		**Number (%)**
Number of patients		45
Sex (male/female)		12/33
Age (mean ± SD), years		45 ± 14 (24–81)
Papillary thyroid cancer		40
	Tumor size (mean ± SD), cm	2.9 ± 1.1 (0.8–5.2)
	Extrathyroidal invasion	28 (70%)
	Multifocality	16 (40%)
	Lymph node metastasis	25 (63%)
	TNM stage	
	Stage 1	24 (60%)
	Stage 2	1 (3%)
	Stage 3	9 (23%)
	Stage 4	6 (15%)
Follicular adenoma		5

### BRAF mutation of thyroid tumors

BRAF mutation was not identified in any of the follicular adenomas and corresponding normal parts of papillary thyroid cancer. About 78% of the papillary thyroid cancers harbored the BRAF mutation (Table 2). Half of the cases with follicular variant of papillary thyroid cancer were positive for BRAF mutation (*P* = 0.213). Papillary cancer with BRAF mutation was significantly associated with a larger tumor size (*P* = 0.045), extrathyroidal invasion (*P* = 0.012), lymph node metastasis (*P* = 0.008), and a higher TNM stage (*P* = 0.044). Age was not associated with BRAF mutation (*P* = 0.437).

**Table 2:** Correlation of BRAF mutation with clinicopathological parameters of papillary thyroid carcinomas

	**BRAF (+) (n = 31)**	**BRAF (-) (n = 9)**	***P *** **value**
Female	23 (74%)	5 (56%)	0.411
Age (years)	46 ± 15	40 ± 7	0.437
Hashimoto’s thyroiditis	4 (13%)	2 (22%)	0.602
Body mass index (kg/m^2^)	25.1 ± 4.2	22.8 ± 1.3	0.482
Body weight (kg)	62 ± 13	62 ± 1	0.725
Tumor size (cm)	3.1 ± 0.9	2.1 ± 1.2	0.045*
Extrathyroidal invasion	25 (81%)	3 (33%)	0.012*
Multifocality	11 (35%)	5 (56%)	0.441
Lymphovascular invasion	9 (29%)	2 (22%)	1.000
Lymph node metastasis	23 (74%)	2 (22%)	0.008*
TNM stage			0.044*
Stage 1	16 (52%)	8 (89%)	
Stage 2	1 (3%)	0 (0%)	
Stage 3	8 (26%)	1 (11%)	
Stage 4	6 (19%)	0 (0%)	

*P value <0.05.

### Detection of tissue CMV DNA using conventional PCR

Since CMV enters the latent phase after a primary infection with its DNA incorporated into the host’s genome, CMV DNA could be found in tissue DNA extracts of thyroid CMV infection. To investigate whether CMV DNA was present in the thyroid tissue samples, DNA extracted from a total of 45 paired tumorous and adjacent non-neoplastic specimens were studied. CMV was not detected by PCR in any of these samples.

### Detection of tissue CMV DNA using real-time PCR assay

**Figure 1:**
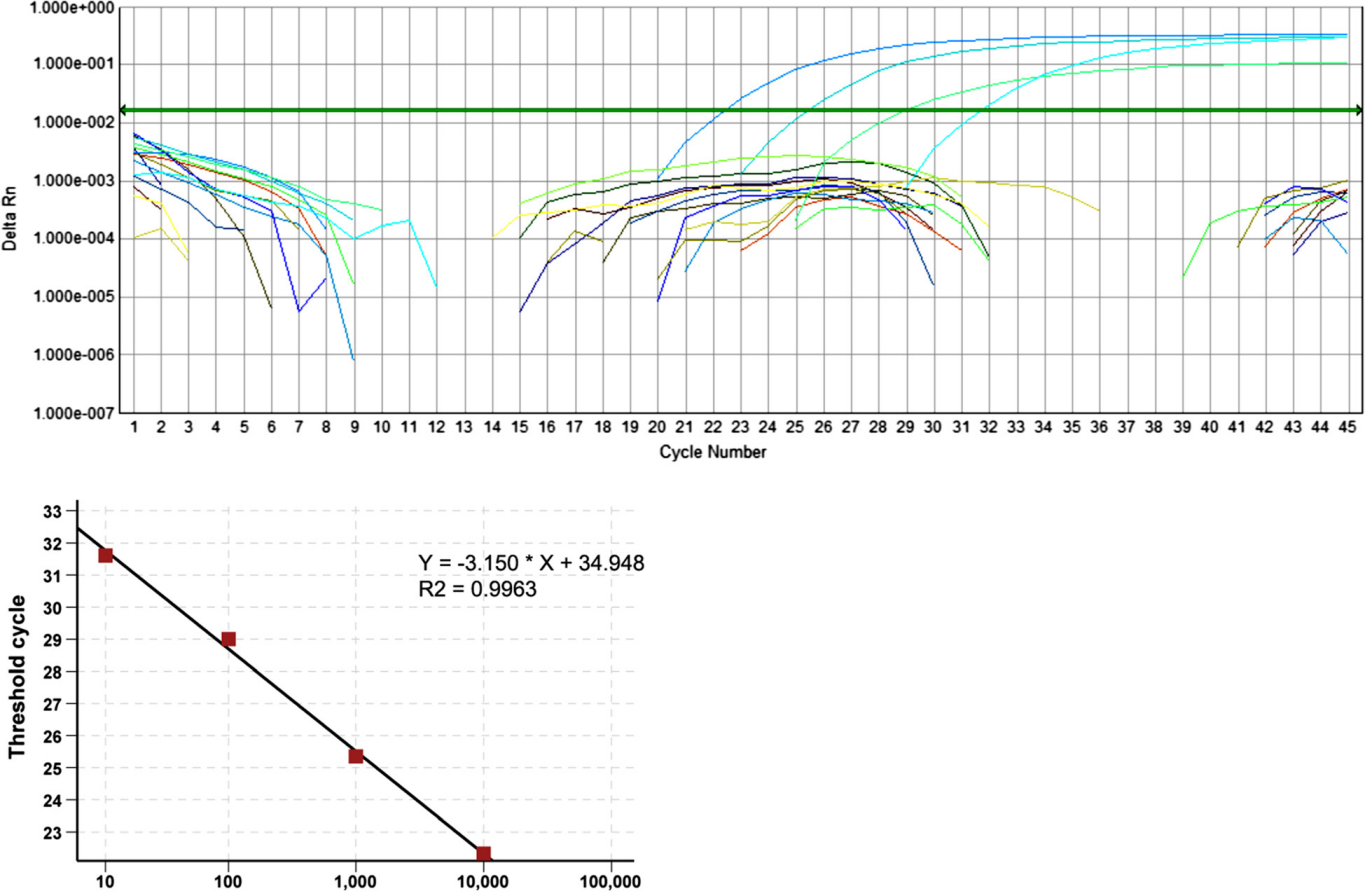
**Real-time quantitative PCR amplification and standard curve of CMV DNA copy numbers.** Upper panel: Threshold cycle (Ct) values are obtained from amplification plots which indicate the change in normalized signal for the four standards between cycles 20 and 40 of the PCR. Ct is the cycle at which fluorescence crosses a threshold value. Lower panel: The standard curve of Ct versus logarithmic DNA copy number. Correlation coefficient is indicated.

To confirm our findings, tissue DNA of thyroid samples was further evaluated using commercial quantitative real-time PCR tests. As shown in Figure 1, there was a strong linear relationship between the threshold cycle (Ct) values and logarithmic DNA inputs. However, no CMV IE DNA could be detected in all tested tissues of follicular adenoma and papillary thyroid cancer.

### Detection of tissue CMV protein using Western blot

**Figure 2:**
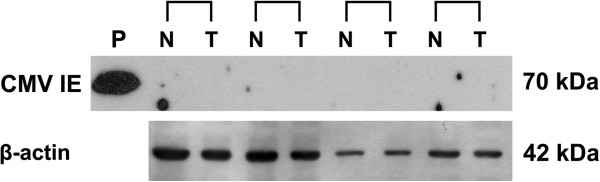
**Detection of tissue CMV protein using Western blot.** Protein levels of CMV immediate-early (IE) antigen were measured by immunoblot analysis in paired papillary thyroid cancer samples (P, positive control; N, normal; T, tumor).

Although no CMV DNA could be found in fresh frozen tissues of follicular adenoma and papillary thyroid cancer, we further determined whether CMV protein was aberrantly expressed in thyroid tumors. In accordance with our aforementioned results, there was no expression of CMV IE protein in 8 pairs of normal and cancerous thyroid tissues (Figure 2).

## Discussion

The link between chronic inflammation and increased risk of developing some cancers is well established [16]. In agreement, thyroid cancer is influenced by and modulates inflammation [17]. Hashimoto’s thyroiditis, one of the most common autoimmune thyroid diseases, is frequently associated with thyroid cancer [18]. Recently, we conducted a population-based cohort study in Taiwan, demonstrating an increased risk for the development of thyroid cancer after a diagnosis of thyroiditis [19]. Thomas et al. [20] examined herpes virus DNA in tissue samples of 4 multinodular goiter and 18 autoimmune thyroid disease (Graves’ disease and Hashimoto’s thyroiditis). They found that the percentage of the presence of at least one kind of herpes virus DNA (herpes simplex virus type 1 and 2, and herpes virus type 6 and 7) is significantly higher in autoimmune thyroid disease than in multinodular goiter (72% vs. 25%). Although the thyroid gland is one of the CMV reservoirs, CMV DNA was not detected in these 22 samples. In other studies, components of several viruses such as hepatitis C virus, human parvovirus B19, Coxsackie virus, and herpes virus could be detected in the thyroid of Hashimoto’s thyroiditis patients [21]. However, at the present time there is insufficient evidence for the viral hypothesis in Hashimoto’s thyroiditis.

Microorganisms causing chronic inflammation have become increasingly investigated as possible cancer initiators/promoters. There has been very little consideration of the potential role of infectious process in the pathogenesis of thyroid cancer, although inflammation has been implicated in the development of thyroid cancer. Herpes simplex virus type 2 was found to be significantly associated with papillary thyroid cancer and the presence of lymph node metastases [22]. Furthermore, human parvovirus B19 has been frequently present in thyroid tissues of Hashimoto’s thyroiditis and papillary thyroid cancer [23]. Although the thyroid gland is one of the CMV reservoirs [12,13], no previous study has examined the presence of CMV in thyroid cancer. The findings of this study suggest that CMV infection is unlikely to be associated with papillary thyroid cancer.

CMV infection often exhibits an altered pattern of IE protein expression. Such proteins act through highly sophisticated mechanisms to facilitate viral production and to avoid detection and elimination of the virus by the immune system [9]. Interestingly, BRAF activation is involved in the expression of CMV IE antigen [24]. Sorafenib is a tyrosine kinase inhibitor being used in advanced iodine-refractory thyroid cancer and is known to inhibit BRAF kinase phosphorylation in the MAPK pathway [25]; of note is the fact that sorafenib also inhibits CMV replication [24]. In our study, about 78% of papillary thyroid cancer harbored the BRAF mutation. Larger tumor size, extrathyroidal invasion, lymph node metastasis, and more advanced TNM stage were associated with the BRAF mutation. This is in keeping with the experience of others [26]. The prevalence of the BRAF mutation in papillary thyroid cancer varies from 32% to 90% in the literature [27], depending on detection methods and histopathological subtypes. Given that the majority of our patients had classic subtype of papillary thyroid cancer, our positive rate of BRAF mutation (78%) was compatible to that reported by other endocrine surgery centers [28].

We recognize some of the limitations of our study. The number of patients studied was small, and results obtained from our selected population may not be extrapolated to other populations. In addition, we did not investigate CMV serological status among our patients. A previous study has shown that CMV DNA could be widely distributed in organs of both seropositive and seronegative healthy individuals [29]. Therefore, we directly assayed the presence of the CMV DNA and protein in the thyroid gland without serological tests.

## Conclusions

CMV DNA and protein were not detected in fresh frozen tissues of follicular adenoma and papillary thyroid cancer, irrespective of the presence or absence of BRAF mutation. The possible role of CMV in the pathogenesis of thyroid cancer is not supported by our study.

## Abbreviations

CMV: Cytomegalovirus; BRAF: v-raf murine sarcoma viral oncogene homolog B; IE: Immediate-early; MAPK: Mitogen-activated protein kinase.

## Competing interests

All authors declare that they have no competing interests.

## Authors’ contributions

TSH and SPC carried out the experiments. TSH, JJL, and SPC conceived of the study, participated in its design and coordination, and helped to draft the manuscript. All authors read and approved the final manuscript.

## References

[B1] Xing M Molecular pathogenesis and mechanisms of thyroid cancer Nat Rev Cancer 2013 13 184 199 10.1038/nrc3431 23429735 PMC3791171

[B2] Vriens MR Suh I Moses W Kebebew E Clinical features and genetic predisposition to hereditary nonmedullary thyroid cancer Thyroid 2009 19 1343 1349 10.1089/thy.2009.1607 20001717

[B3] Dal Maso L Bosetti C La Vecchia C Franceschi S Risk factors for thyroid cancer: an epidemiological review focused on nutritional factors Cancer Causes Control 2009 20 75 86 10.1007/s10552-008-9219-5 18766448

[B4] Caronia LM Phay JE Shah MH Role of BRAF in thyroid oncogenesis Clin Cancer Res 2011 17 7511 7517 10.1158/1078-0432.CCR-11-1155 21900390

[B5] Melillo RM Castellone MD Guarino V De Falco V Cirafici AM Salvatore G Caiazzo F Basolo F Giannini R Kruhoffer M Orntoft T Fusco A Santoro M The RET/PTC-RAS-BRAF linear signaling cascade mediates the motile and mitogenic phenotype of thyroid cancer cells J Clin Invest 2005 115 1068 1081 10.1172/JCI200522758 15761501 PMC1062891

[B6] Liu CL Lee JJ Liu TP Chang YC Hsu YC Cheng SP Blood neutrophil-to-lymphocyte ratio correlates with tumor size in patients with differentiated thyroid cancer J Surg Oncol 2013 107 493 497 10.1002/jso.23270 22996403

[B7] Griffiths PD Burden of disease associated with human cytomegalovirus and prospects for elimination by universal immunisation Lancet Infect Dis 2012 12 790 798 10.1016/S1473-3099(12)70197-4 23017365

[B8] Cannon MJ Schmid DS Hyde TB Review of cytomegalovirus seroprevalence and demographic characteristics associated with infection Rev Med Virol 2010 20 202 213 10.1002/rmv.655 20564615

[B9] Soderberg-Naucler C HCMV microinfections in inflammatory diseases and cancer J Clin Virol 2008 41 218 223 10.1016/j.jcv.2007.11.009 18164235

[B10] Johnsen JI Baryawno N Soderberg-Naucler C Is human cytomegalovirus a target in cancer therapy? Oncotarget 2011 2 1329 1338 22246171 10.18632/oncotarget.383 PMC3282090

[B11] Reeves MB Breidenstein A Compton T Human cytomegalovirus activation of ERK and myeloid cell leukemia-1 protein correlates with survival of latently infected cells Proc Natl Acad Sci USA 2012 109 588 593 10.1073/pnas.1114966108 22203987 PMC3258610

[B12] Tsai JH Tsai CH Cheng MH Lin SJ Xu FL Yang CC Association of viral factors with non-familial breast cancer in Taiwan by comparison with non-cancerous, fibroadenoma, and thyroid tumor tissues J Med Virol 2005 75 276 281 10.1002/jmv.20267 15602723

[B13] Chen T Hudnall SD Anatomical mapping of human herpesvirus reservoirs of infection Mod Pathol 2006 19 726 737 10.1038/modpathol.3800584 16528368

[B14] Ranganathan P Clark PA Kuo JS Salamat MS Kalejta RF Significant association of multiple human cytomegalovirus genomic loci with glioblastoma multiforme samples J Virol 2012 86 854 864 10.1128/JVI.06097-11 22090104 PMC3255835

[B15] Cheng SP Liu CL Hsu YC Chang YC Huang SY Lee JJ Expression and biologic significance of adiponectin receptors in papillary thyroid carcinoma Cell Biochem Biophys 2013 65 203 210 10.1007/s12013-012-9419-1 22907586

[B16] Grivennikov SI Greten FR Karin M Immunity, inflammation, and cancer Cell 2010 140 883 899 10.1016/j.cell.2010.01.025 20303878 PMC2866629

[B17] Guarino V Castellone MD Avilla E Melillo RM Thyroid cancer and inflammation Mol Cell Endocrinol 2010 321 94 102 10.1016/j.mce.2009.10.003 19835928

[B18] Ye ZQ Gu DN Hu HY Zhou YL Hu XQ Zhang XH Hashimoto’s thyroiditis, microcalcification and raised thyrotropin levels within normal range are associated with thyroid cancer World J Surg Oncol 2013 11 56 10.1186/1477-7819-11-56 23496874 PMC3717052

[B19] Liu CL Cheng SP Lin HW Lai YL Risk of thyroid cancer in patients with thyroiditis: a population-based cohort study Ann Surg Oncol 2014 21 843 849 10.1245/s10434-013-3363-1 24201747

[B20] Thomas D Liakos V Michou V Kapranos N Kaltsas G Tsilivakos V Tsatsoulis A Detection of herpes virus DNA in post-operative thyroid tissue specimens of patients with autoimmune thyroid disease Exp Clin Endocrinol Diabetes 2008 116 35 39 10.1055/s-2007-956171 18240111

[B21] Mori K Yoshida K Viral infection in induction of Hashimoto’s thyroiditis: a key player or just a bystander? Curr Opin Endocrinol Diabetes Obes 2010 17 418 424 10.1097/MED.0b013e32833cf518 20625285

[B22] Jensen K Patel A Larin A Hoperia V Saji M Bauer A Yim K Hemming V Vasko V Human herpes simplex viruses in benign and malignant thyroid tumours J Pathol 2010 221 193 200 10.1002/path.2701 20455254

[B23] Adamson LA Fowler LJ Clare-Salzler MJ Hobbs JA Parvovirus B19 infection in Hashimoto’s thyroiditis, papillary thyroid carcinoma, and anaplastic thyroid carcinoma Thyroid 2011 21 411 417 10.1089/thy.2010.0307 21190433

[B24] Michaelis M Paulus C Loschmann N Dauth S Stange E Doerr HW Nevels M Cinatl J Jr The multi-targeted kinase inhibitor sorafenib inhibits human cytomegalovirus replication Cell Mol Life Sci 2011 68 1079 1090 10.1007/s00018-010-0510-8 20803231 PMC11114814

[B25] Marotta V Ramundo V Camera L Del Prete M Fonti R Esposito R Palmieri G Salvatore M Vitale M Colao A Faggiano A Sorafenib in advanced iodine-refractory differentiated thyroid cancer: efficacy, safety and exploratory analysis of role of serum thyroglobulin and FDG-PET Clin Endocrinol (Oxf) 2013 78 760 767 10.1111/cen.12057 23009688

[B26] Kim YS Kim JS Bae JS Park WC Clinical implication of the BRAFV600E mutation in papillary thyroid carcinoma World J Surg Oncol 2013 11 99 10.1186/1477-7819-11-99 23687957 PMC3660263

[B27] Jeong D Jeong Y Park JH Han SW Kim SY Kim YJ Kim SJ Hwangbo Y Park S Cho HD Oh MH Yang SH Kim CJ BRAF (V600E) mutation analysis in papillary thyroid carcinomas by peptide nucleic acid clamp real-time PCR Ann Surg Oncol 2013 20 759 766 10.1245/s10434-012-2494-0 23179992

[B28] Li C Aragon Han P Lee KC Lee LC Fox AC Beninato T Thiess M Dy BM Sebo TJ Thompson GB Grant CS Giordano TJ Gauger PG Doherty GM Fahey TJ 3rd Bishop J Eshleman JR Umbricht CB Schneider EB Zeiger MA Does BRAF V600E mutation predict aggressive features in papillary thyroid cancer? Results from four endocrine surgery centers J Clin Endocrinol Metab 2013 98 3702 3712 10.1210/jc.2013-1584 23969188

[B29] Hendrix RM Wagenaar M Slobbe RL Bruggeman CA Widespread presence of cytomegalovirus DNA in tissues of healthy trauma victims J Clin Pathol 1997 50 59 63 10.1136/jcp.50.1.59 9059359 PMC499715

